# Potential distribution of *Biscogniauxia mediterranea and Obolarina persica* causal agents of oak charcoal disease in Iran’s Zagros forests

**DOI:** 10.1038/s41598-024-57298-2

**Published:** 2024-04-02

**Authors:** Meysam BakhshiGanje, Shirin Mahmoodi, Kourosh Ahmadi, Mansoureh Mirabolfathy

**Affiliations:** 1 Kohgiluyeh va Boyer-Ahmad Agricultural and Natural Resources Research and Education Center, Yasuj, Iran; 2 National center of genetic resources, Agricultural Research Education and Extention Organization, Tehran, Iran; 3 Department of Forestry, Faculty of Natural Resources and Marine Sciences, Tarbiat Modares University, Tehran, Iran; 4 Fars Agricultural and Natural Resources Research and Education Center (AREEO), Tehran, Iran; 5 Iranian Research Institute of Plant Protection, Tehran, Iran

**Keywords:** Oak decline, Charcoal disease, Zagros forests, Machine-learning, Species distribution models, Iran, Climate change, Agroecology, Ecological modelling, Microbial ecology

## Abstract

In Iran, native oak species are under threat from episodes of Charcoal Disease, a decline syndrome driven by abiotic stressors (e.g. drought, elevated temperature) and biotic components, *Biscogniauxia mediterranea* (De Not.) Kuntze and *Obolarina persica* (M. Mirabolfathy). The outbreak is still ongoing and the country’s largest ever recorded. Still, the factors driving its’ epidemiology in time and space are poorly known and such knowledge is urgently needed to develop strategies to counteract the adverse effects. In this study, we developed a generic framework based on experimental, machine-learning algorithms and spatial analyses for landscape-level prediction of oak charcoal disease outbreaks. Extensive field surveys were conducted during 2013–2015 in eight provinces (more than 50 unique counties) in the Zagros ecoregion. Pathogenic fungi were isolated and characterized through morphological and molecular approaches, and their pathogenicity was assessed under controlled water stress regimes in the greenhouse. Further, we evaluated a set of 29 bioclimatic, environmental, and host layers in modeling for disease incidence data using four well-known machine learning algorithms including the Generalized Linear Model, Gradient Boosting Model, Random Forest model (RF), and Multivariate Adaptive Regression Splines implemented in MaxEnt software. Model validation statistics [Area Under the Curve (AUC), True Skill Statistics (TSS)], and Kappa index were used to evaluate the accuracy of each model. Models with a TSS above 0.65 were used to prepare an ensemble model. The results showed that among the different climate variables, precipitation and temperature (Bio18, Bio7, Bio8, and bio9) in the case of *O. persica* and similarly, gsl (growing season length TREELIM, highlighting the warming climate and the endophytic/pathogenic nature of the fungus) and precipitation in case of *B. mediterranea* are the most important influencing variables in disease modeling, while near-surface wind speed (sfcwind) is the least important variant. The RF algorithm generates the most robust predictions (ROC of 0.95; TSS of 0.77 and 0.79 for MP and OP, respectively). Theoretical analysis shows that the ensemble model (ROC of 0.95 and 0.96; TSS = 0.79 and 0.81 for MP and OP, respectively), can efficiently be used in the prediction of the charcoal disease spatiotemporal distribution. The oak mortality varied ranging from 2 to 14%. Wood-boring beetles association with diseased trees was determined at 20%. Results showed that water deficiency is a crucial component of the oak decline phenomenon in Iran. The Northern Zagros forests (Ilam, Lorestan, and Kermanshah provinces) along with the southern Zagros forests (Fars and Kohgilouyeh va-Boyer Ahmad provinces) among others are the most endangered areas of potential future pandemics of charcoal disease. Our findings will significantly improve our understanding of the current situation of the disease to pave the way against pathogenic agents in Iran.

## Introduction

Forests cover approximately one-third of the global landscape and provide essential ecological, economic and cultural services, including carbon sequestration and primary production, which are central to mitigating the impacts of climate change^1^. However, climate change is significantly impacting the health of forest biomes, driving abiotic stressors of plant health and exacerbating the risk of devastating tree diseases caused by insect pests and pathogens. This expectation is based on the likelihood that the environmental stress on trees will increase, (e.g., due to more frequent and intensive drought and heat episodes) and as changing temperature and precipitation regimes, coupled with pest and pathogen introductions via the global plant trade, will allow pests and pathogens to thrive in geographic regions where they previously could not survive, e.g., because of cold temperatures^2,3^.

**Figure 1:**
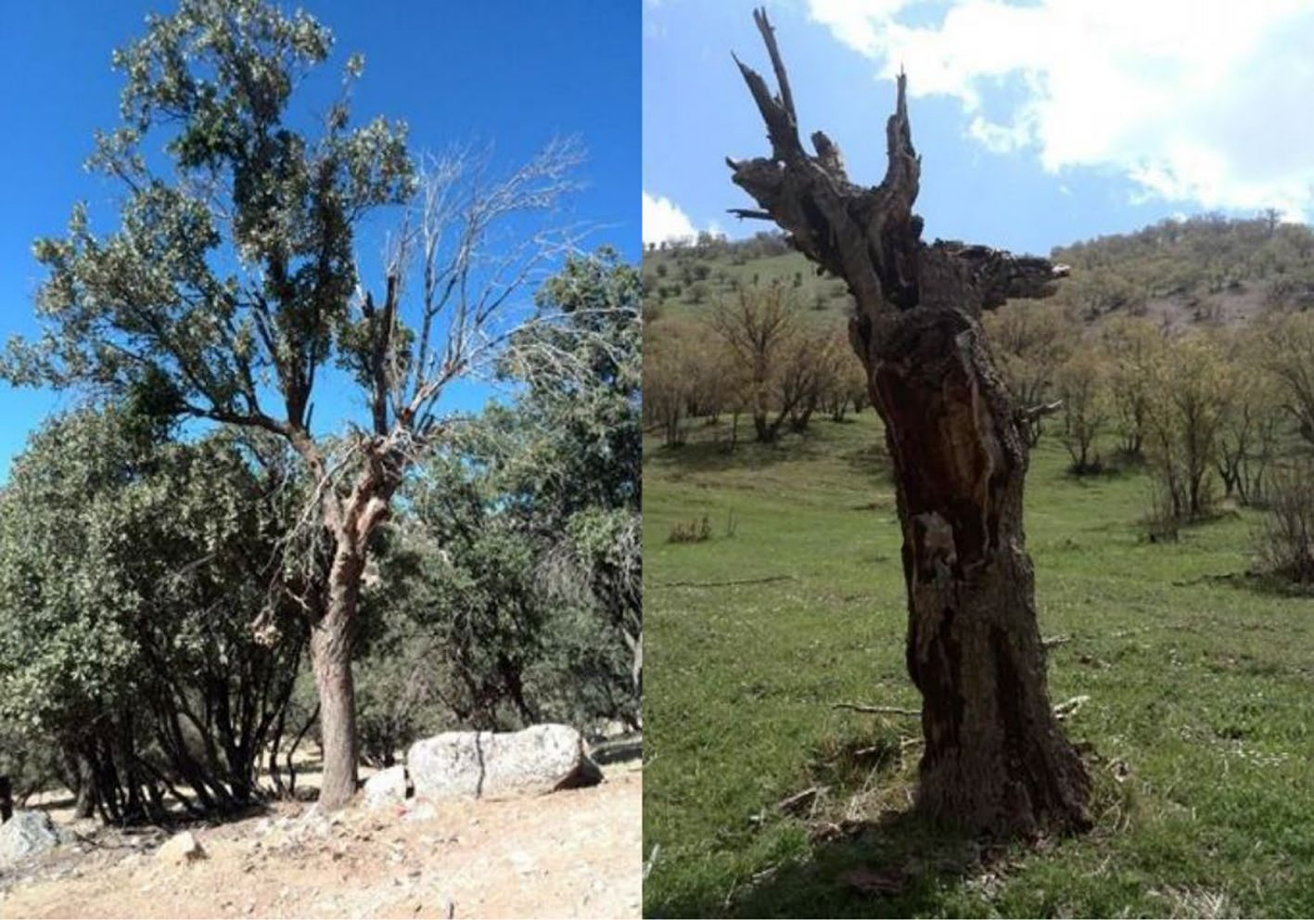
Complete (right) and one-sided drying (left) of the oak tree due to infection of the trunk and crown by the oak charcoal disease.

Oak trees (genus *Quercus*) are important to deciduous forests globally and are increasingly threatened by abiotic and biotic stressors^4^. The oak decline is characterized by periodic occurrences of decline and death of oaks over widespread areas^5^. In a comprehensive review, Pourhashemi and Sadeghi^6^ described multidimensional, complex and sporadic aspects of research conducted on Iranian oak tree decline. Besides the conflicting results in the relationship between decline and quantitative characteristics of trees, the clumping pattern of declined trees and the direct relationship between stand density and syndrome with relative certainty has been noticed and definite evidence of a positive correlation between decline syndrome and soil properties has been highlighted (severe decline symptoms in shallow soils in comparison to deep soils). Among the biotic stressors, fungal, and bacterial pathogens in addition to wood-boring beetles (*Buprestidae* and *Cerambycidae*) and some cases oomycetes (*Phytophthora* sp., *Pythium* sp.), are recognized as the most prevalent agents associated with diseased oak trees in Iran^5,6^. The charcoal disease epidemics (caused by *Biscogniauxia mediterranea* (De Not.) Kuntze and *Obolarina persica* [M. Mirabolfathy]), have been characterized widely in Iran. The decline began with browning of the leaves, viscous liquid exaltation on the branches and trunks resulting in a brown-black discoloration of bark and woody tissues^7,8^ (Fig. 1).

Species distribution models (SDMs) are now widely used across different ecosystems to predict species response to climate change^9^. Beyond predicting species distribution, these models have become an important decision-making tool for a variety of ecogeographical applications, such as identifying potential conservation areas, determining potential locations for the distribution of sensitive and invasive species, and mapping the spread of forest disease. These types of modeling, by combining separate environmental factors with spatial data of species distribution, with the help of statistical models such as Generalized Linear Model (GLM) and Gradient Boosting Model (GBM) among others, and by using machine learning methods, seek to find regular and logical relationships between species distribution and different variables^10–12^.

As a result of climate change, predisposing factors like successive droughts, temperature, and humidity are thought to be the major attributed factors implicated in the decline of oak worldwide^3,4,7,8,13–26^. The present study aimed at epidemiological modeling of the charcoal disease on oak forests in the Zagros habitat, west of Iran. We estimate that the areas prone to epidemic development can be predicted by a combination of massive on-site data analysis and machine-learning approaches. Data collected on the affected trees in an area of 3.1 M ha of oak forests and different bioclimatic/environmental variables and host distribution data were implemented in disease predictive modeling. Regions with a high chance of disease incidence and progression were predicted. To our knowledge, the first holistic view of the oak charcoal disease modeling using five machine-learning algorithms is presented here and could help to adopt disease management strategies against pathogenic agents and find trends and mechanisms in fungal pathogens adaptation and distribution in uncertain environmental futures due to climate change.

## Materials and methods

### Study area

**Figure 2:**
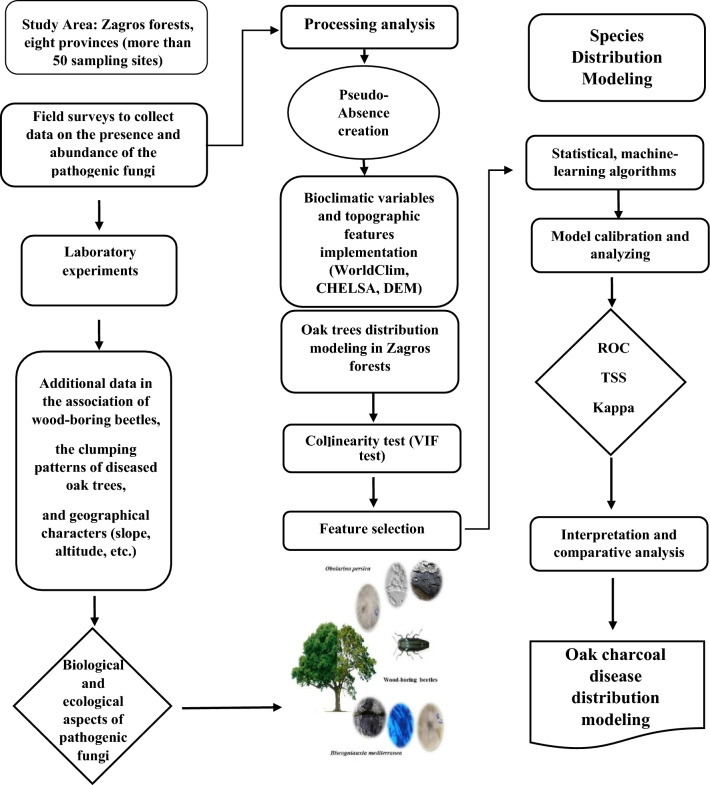
Research work plan. The flow chart represents the methodology and modeling procedure of oak charcoal disease distribution.

**Figure 3:**
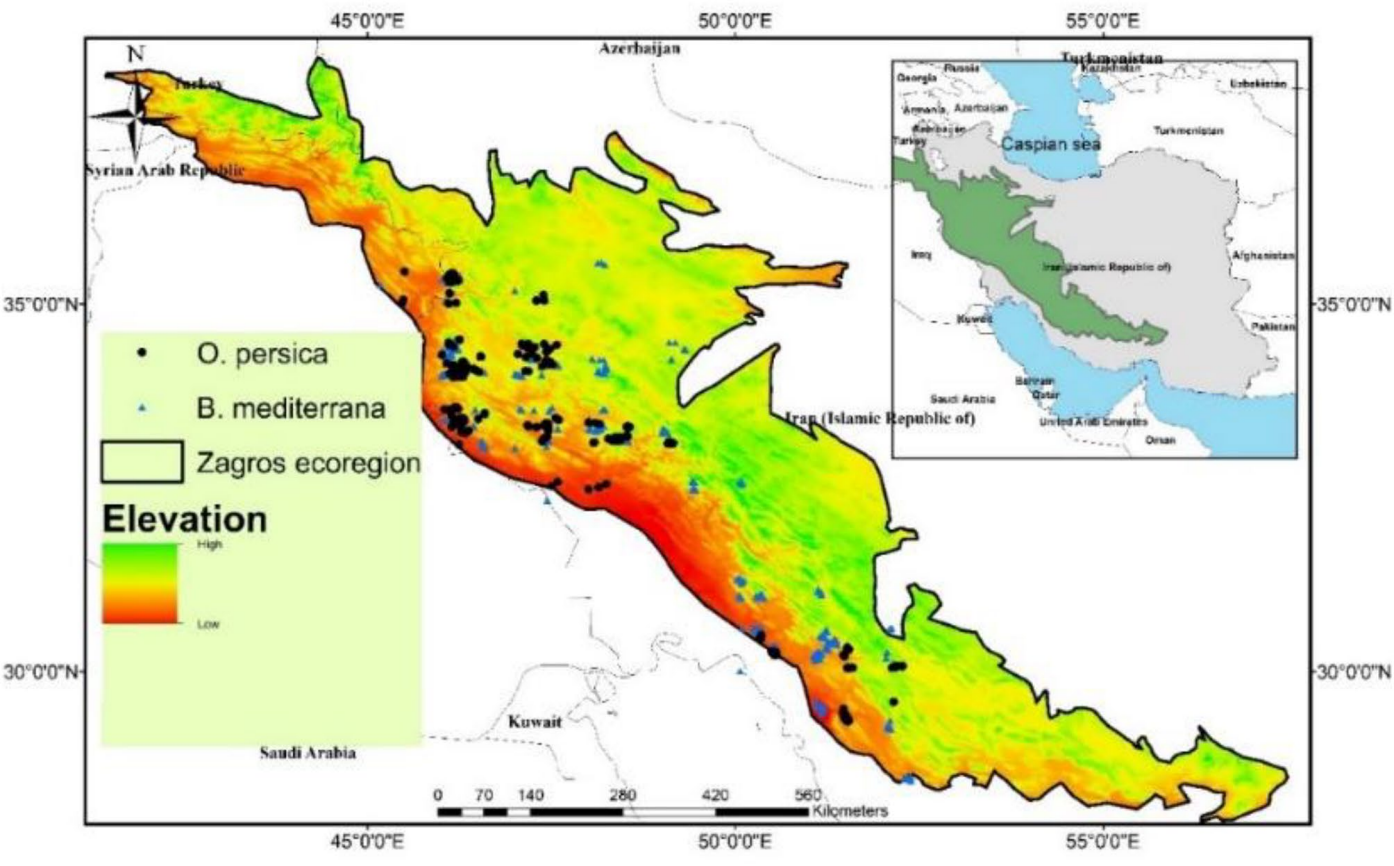
The study area. The green polygons (top right) approximately refer to the Zagros ecoregion (Doubek 2013).

With almost 14.3 million hectares, the Iranian forests are located in the north and northwestern to the southwestern of the plateau. The survey summary is presented in Fig. 2. Healthy and suspected oak trees affected by CD were monitored (from spring to autumn in three successive years from 2013 to 2015) across Zagros forests (over 3M ha), including Ilam, Kermanshah, Hamedan, Lorestan, Kougilouyeh va Boyer-Ahmad, Fars, Khuzestan and Kurdistan (Marivan county) provinces located in Zagros mountains (Fig. 3). Also, over 20 hectares of experimental plots localized in Ilam, Kermanshah, and Kougilouyeh va Boyer-Ahmad provinces were selected aimed at screening the biological aspects of the CD causal agents, its local distribution, increase in the number of affected trees during the assessments and changes in tree health status. Experimental and field assessments including charcoal disease etiological assessments have been provided for all locations in the Supplementary material.

### Data collection

#### Field survey and tree health assessment

The attributes of infected areas were documented including the biotic/abiotic stressors, the position, the dominant type of host tree species, the tree in the high forest and scrub forms, health appearance, association of wood-boring beetles with diseased trees, and the evidence of current and previous diseases incidence.

#### Determination of the host and pathogen’s occurrence locations

A field survey to assess the occurrence of CD was conducted. From each site, at least an area of 1 km^2^ was randomly inspected for a disease incidence survey. The height digital model variable with a cell size of one square kilometer was chosen as an important elevation variable in the sensitivity modeling of oak forests^27^. Also, this variable was used to prepare the slope variable in the Spatial Analyst tool in the ArcGIS 10.3 software environment. The sensitivity map of oak forests to fungal pathogens (BM and OP) in the study area was prepared using the Biomod2 package in the R version 3.6 ^28^. Over two thousand sampling points of presence records of the pathogenic fungi were collected. In addition to presence records, pseudo‐absence points were obtained with the random point method using GIS software.

#### Bioclimatic/environmental variables

Initially, 29 bioclimatic/environmental variables that may affect the species distribution were considered. They include growing season-length TREELIM (Gsl, number of days), The mean temperature of the growing season TREELIM (Gst, °C), Near-surface relative humidity (Hurs, %), Potential evapotranspiration (Pet, kg m^−2^ month^−1^), Near-surface wind speed (Sfcwind, m s^−1^), Accumulated precipitation amount on (Gsp, kg m^−2^ gsl^−1^), Climate moisture index (CMI, kg m^−2^ month^−1^), Annual mean temperature (Bio1, °C), Mean Diurnal Range (Mean of monthly (max temp–min temp) (Bio2, °C), Isothermality (BIO2/BIO7) (× 100) (Bio3, °C), Temperature Seasonality (standard deviation × 100) (Bio4, °C), Max Temperature of Warmest Month (Bio5, °C), Min Temperature of Coldest Month (Bio6, °C), Temperature Annual Range (BIO5-BIO6) (Bio7, °C), Mean Temperature of Wettest Quarter (Bio8, °C ), Mean Temperature of Driest Quarter (Bio9, °C), Mean Temperature of Warmest Quarter (Bio10, °C), Mean Temperature of Coldest Quarter (Bio11, °C), Max Temperature of Warmest Month (Bio12, °C), Precipitation of Wettest Month (Bio13, mm), Precipitation of Driest Month (Bio14, mm), Precipitation Seasonality (Coefficient of Variation) (Bio15, mm), Precipitation of Wettest Quarter (Bio16, mm), Precipitation of Driest Quarter (Bio17, mm), Precipitation of Warmest Quarter (Bio18, mm), Precipitation of Coldest Quarter (Bio19, mm)^29–31^ following with Slope (%) and Solar-radiation Aspect Index, derived from The SRTM (Shuttle Radar Topography Mission data) aimed to prepare the digital elevation model (DEM)^27,32,33^.

Considering possible errors due to the high correlation between the environmental variables and their consequence effects on model accuracy, the correlations between the selected variables were determined using the variance inflation factor (VIF). The equation is described in the following equation: *Tolerance* = 1 − *Rj*^2^, *VIF* = 1/*Toleranc.*

In this regard, *Rj*^2^ refers to the coefficient of determination (R Square) of the regression model. Finally, 14 out of 29 bioclimatic variables (bold variables in Table 1), which were slightly correlated with each other were selected for model processing including Gsl, Gst, Hurs, Pet, Sfcwind, CMI, Bio3, Bio7, Bio8**,** Bio9, Bio13, Bio14, Bio15, and Bio18, in addition to the host oak trees coverage percentage. The provided layers were transformed into ASCII format for subsequent processing in MaxEnt software^31–36^.

**Table 1:** Bioclimatic layers used for predictive oak charcoal disease modeling.

Type	Abbreviations	Description	Unit	Source
Bioclimatic	Gsl	Growing season-length TREELIM	Number of days	https://chelsa-climate.org^31^
	Gst	The mean temperature of the growing season TREELIM	°C	
	Hurs	Near-surface relative humidity	%	
	Pet	Potential evapotranspiration	kg m^−2^ month^−1^	
	Sfcwind	Near-surface wind speed	m s^−1^	
	Gsp	Accumulated precipitation amount on	kg m^−2^ gsl^−1^	
	Cmi	Climate moisture index	kg m^−2^ month^−1^	
	Bio1	Annual mean temperature	°C	www.worldclim2.org^30^
	Bio2	Mean diurnal range (mean of monthly (max temp–min temp)	°C	
	Bio3	Isothermality (BIO2/BIO7) (× 100)	°C	
	Bio4	Temperature seasonality (standard deviation × 100)	°C	
	Bio5	Max temperature of warmest month	°C	
	Bio6	Min temperature of coldest month	°C	
	Bio7	Temperature annual range (BIO5-BIO6)	°C	
	Bio8	Mean temperature of wettest quarter	°C	
	Bio9	Mean temperature of driest quarter	°C	
	Bio10	Mean Temperature of Warmest Quarter	°C	
	Bio11	Mean temperature of coldest Quarter	°C	
	Bio12	Max temperature of warmest month	°C	
	Bio13	Precipitation of wettest month	mm	
	Bio14	Precipitation of driest month	mm	
	Bio15	Precipitation seasonality (coefficient of variation)	mm	
	Bio16	Precipitation of wettest quarter	mm	
	Bio17	Precipitation of driest quarter	mm	
	Bio18	Precipitation of warmest quarter	mm	
	Bio19	Precipitation of coldest quarter	mm	
Topography	SLP	Slope	%	(DEM) (https://earthexplorer.usgs.gov/)
	TRASP	Solar-radiation aspect index	(kJ m^−2^ day^−1^)	^30^
Host	Tree cover		%	

### Data analysis

#### Species distribution modeling

Generalized Linear Model (GLM)^36^, Generalized Boosting Model (GBM)^37^, Multivariate Adaptive Regression Splines (MARS)^38^, and Random Forest (RF)^39^ were used to determine the sensitivity map of oak forests to these fungal pathogens. The ensemble model is a powerful approach that increases the accuracy of the model by combining different models^40,41^. The Biomod2 package in R environment has used this approach and by calculating the weighted average of predictive models, prepares a consensus map of habitat distribution^42^. The spatial resolution of bioclimatic variables was at 30 arc-seconds (~ 1 km). Bioclimatic layers’ clipping was performed in ArcGIS 10.3. Modeling was done for each model in 10 repetitions. 75% of the presence and background points were considered as educational data and the remaining 25% as test data^43^. According to the need of the mentioned models for background data, the number of 1000 pseudo-missing points was randomly considered in the studied area and outside the two-kilometer radius of the presence points.

#### Models validation

The area under the curve (AUC), the true skill statistic (TSS), and Kappa were used to evaluate the validity and quality of each model and consensus model^44^. The predictive performances and contributions of all the selected variables were determined using the jackknife test^31,32^. The response curves which indicated the relationship of disease presence points to all single environmental variables were determined. AUC value closer to 1.0 indicates a successful model with clear distinction while an AUC value closer to 0.5 reflects a model with no clear distinction^45,46^. Finally, the consensus map of the sensitivity distribution of diseased oak trees was prepared from the weighted average of the values of the five mentioned models.

## Results

### Field survey

Overall, throughout the northwest to southwest, the CD has spread rapidly in the forests of the Zagros (Supplementary material section). Here for the first time, the occurrence of charcoal diseases has been documented in Isfahan (Fereydoun-Shahr city, Khosh-miveh village and Poshtkouh mougooyi) and Khorasan-Shomali (Ashkhaneh, Darkesh and Cheshme-Goulak forest) provinces, out of Zagros forests. These data were not used in modeling algorithms. The mortality rate in the study areas, depending on the nature of the trees- high forest or coppice forms, was 2–14% between different provinces. The outbreak of the disease was seen more in Ilam province, but disease incidence was higher in Kermanshah province. The highest mortality was observed in the Fars, Lorestan and Ilam provinces, respectively. BM generally had a wider range of distribution in comparison to OP. Results showed that the effect of drought, as the primary cause of tree weakness, is undeniable. No signs of disease were detected on the other forest trees in Zagros’ habitat. Wood-boring beetles (*Cerambycidae* and *Buprestidae*; *Chalcophorella bagdadiensis, Agrilus hastulifer*, *Chrysobotris parvipuncta*) were associated with symptoms of CD in only 20% of cases, which was well evident in Chogha Sabz Forest Park, Ilam in 2015. The symptoms of the disease have not been detected in the root so far. The rapid development of the disease and, as a result, the earlier occurrence of symptoms, occurs in areas where the roots of trees are more exposed to water scarcity and evaporation and transpiration are higher in them. In other words, they are located in marginal forests or ecotones. While the development of the disease is slow in the endophyte stage of the pathogens, the symptoms of the disease appear later on oaks in heartwood depth. The progression of the disease occurs faster in hot and dry plains and on steep and rocky slopes with shallow soil depth. From the onset of infection to its spread and complete drying of infected bases, a period of nearly 2 years has been recorded. Based on the assessment of the current situation, untimely flooding rains and subsequent continuous droughts during the growing season have increased the outbreak of the disease in infected areas, a clear example which was seen in Firooz abad Fars. Conversely, suitable winter rainfall had a favorable effect on the recovery of diseased trees and the production of abundant branches, especially in cases where tending operations including cleaning, lighting, thinning, and realizing were performed.

Persian oak (*Q. brantii* Lindl.) which is considered the dominant oak species in Zagros forests, comprises the majority of surveyed oaks. The colonization of the oak tissues on twigs and stems by the fungal agent was slow in 20–25 year-old trees resulting in gradual development of the pathogen, while it was rapid in the young trees. Most of the trees that were infected on their trunks showed a decline in the upper parts. In all areas, the trees located in the valleys were less infected, compared to those located on the slope, which can probably be attributed to the low availability of water for the roots and the shallow depth of the permeable soil. Callus tissue which shows a kind of resistance mechanism was observed in tree trunks. In some cases, two or more trees of the same species were located very close to each other and showed neither infected tissues nor decline symptoms with vigorous appearance among the rest of the diseased trees which can be selected as the resistance source.

### Variable importance

**Figure 4:**
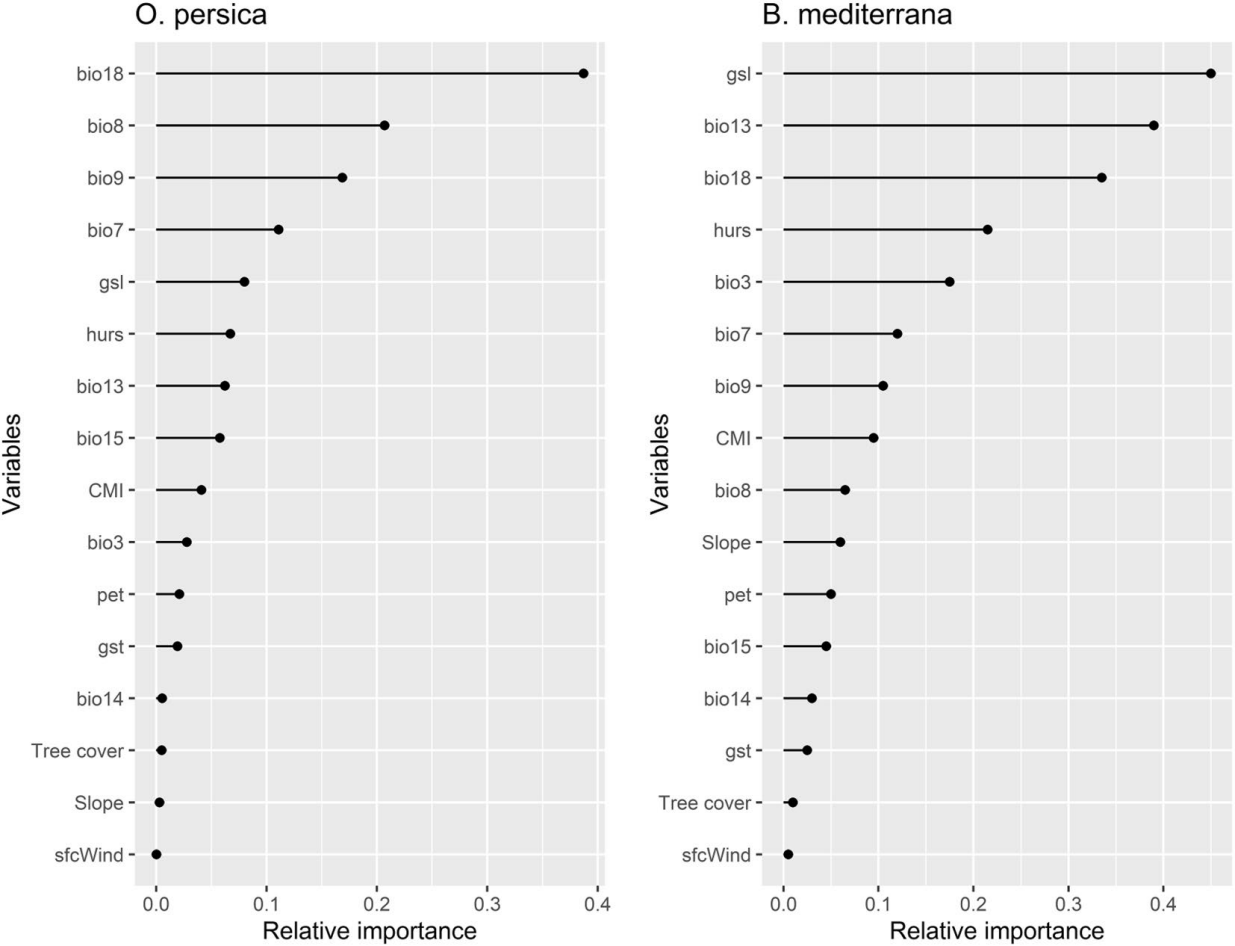
Relative variable importance based on jackknife test training gain.

Variables predictive performances using the jackknife test revealed that the gsl (growing-season length TREELIM) and Bio18 (Precipitation of Warmest Quarter) are the most influential environmental/bioclimatic variables in the distribution and determining the habitat of the BM and OP, respectively. In contrast, Near-surface wind speed precipitation seasonality (sfcwind) is the least important variant with a training gain of zero as presented in Fig. 4 (Table 1).

### Response curve analysis

**Figure 5:**
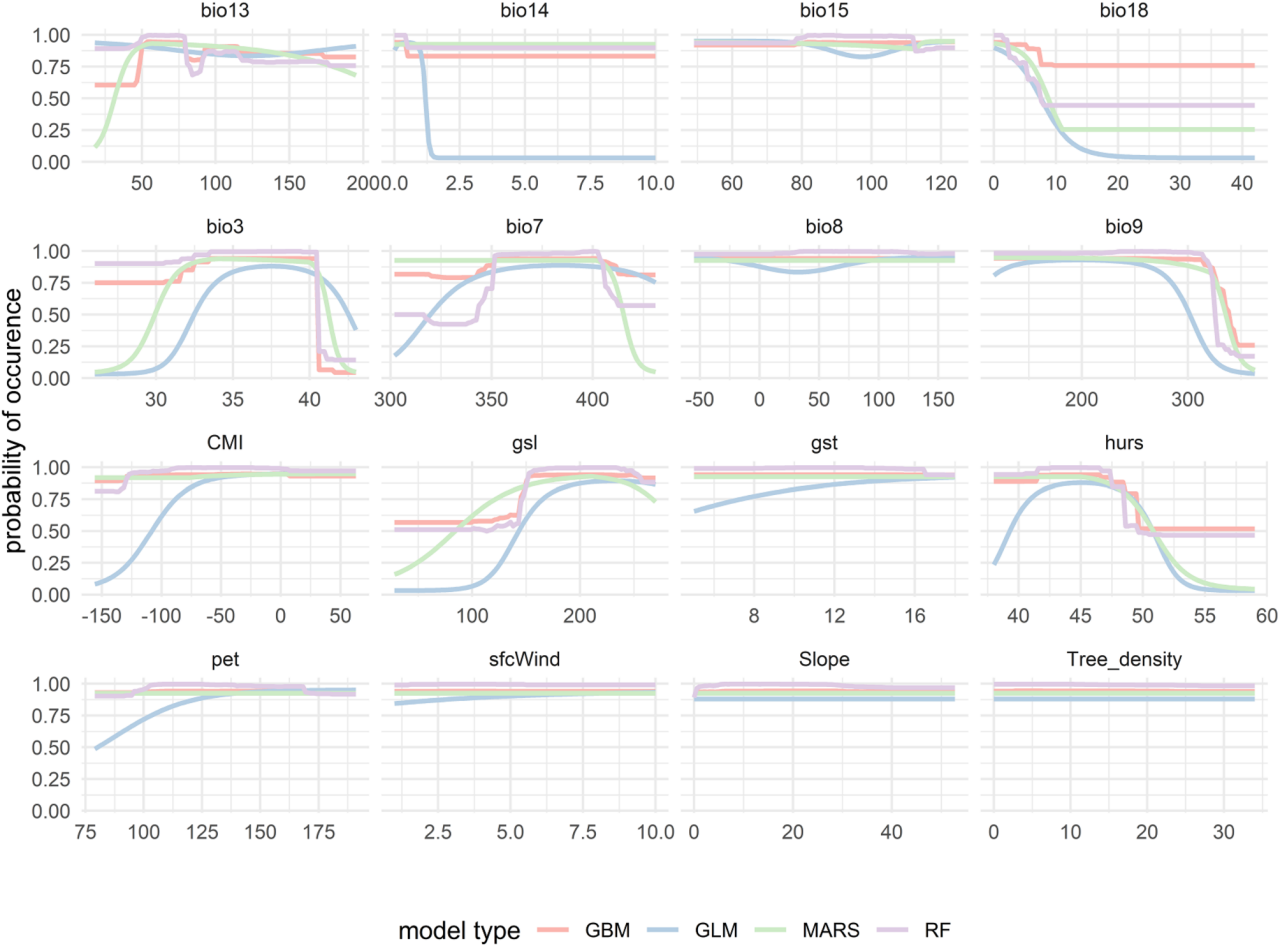
Response curve analysis for the major predictors of suitability habitats of *Biscogniauxia mediterrana* in the study area modeled by Generalized Linear Model (GLM), Multivariate Adaptive Regression Splines (MARS), Generalized Boosting Model (GBM), and Random Forest (RF).

**Figure 6:**
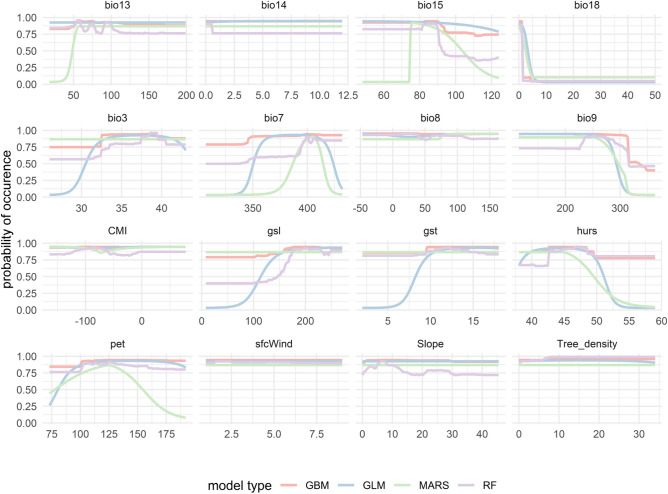
Response curve analysis for the major predictors of suitability habitats of *Obolarina persica* in the study area modeled by Generalized Linear Model (GLM), Multivariate Adaptive Regression Splines (MARS), Generalized Boosting Model (GBM), and Random Forest (RF).

According to the response curves, higher probabilities of the pathogenic fungi presence are obtained in determined values of variables. Almost even a slope increase of occurrence in the study area was observed in BM, however, with increases in bio3 and bio7, bio9, and hurs, the probability of species presence drastically shows a decrease. In addition, with a primary decrease of bio18, the species presence follows almost an even pattern (Fig. 5). OP response curves demonstrated that except for bio7, bio9, bio15, bio18, and hurs, which show a decreasing species occurrence with a sharp pattern, other environmental variables show an increase in probabilities (Fig. 6).

### Performance evaluation of models

The results of the evaluation of species distribution models are shown in Tables 2 and 3. With an ROC of up to 96, the species habitat map efficiently determines the habitat suitability distribution. RF shows a very good performance in predicting the species distribution of both BM and OP (ROC = 0.95), very close to the ensemble model. The value of TSS indicates the good accuracy of the ensemble model in the species distribution of OP (TSS = 0.95) in comparison to TSS = 79 for BM distribution. According to the Kappa index, ensemble models are suitable models for predicting the species distribution of BM and OP compared to the rest of the models (Tables 2 and 3).

**Table 2:** Evaluation of species distribution models of *B. mediterrana* potential distribution.

Evaluation criteria	Models				
	RF	GBM	GLM	MARS	Ensemble model
TSS	0.77	0.66	0.56	0.61	0.79
ROC	0.95	0.88	0.83	0.84	0.96
Kappa	0.75	0.62	0.55	0.58	0.77

**Table 3:** Evaluation of species distribution models of *O. persica* potential distribution.

Evaluation criteria	Model				
	RF	GBM	GLM	MARS	Ensemble model
TSS	0.796	0.736	0.652	0.756	0.81
ROC	0.949	0.931	0.881	0.893	0.95
Kappa	0.76	0.68	0.57	0.64	0.77

### *B. mediterrana *and *O. persica* distributions in the study area

**Figure 7:**
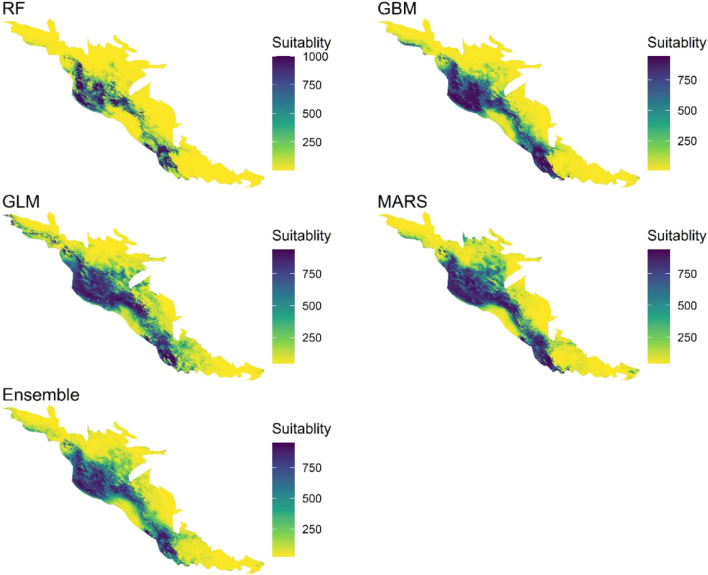
Model predictions of suitability generated using *B. mediterrana* dataset at 1 km square scale in the west forests of Iran. Area modeled by Generalized Linear Model (GLM), Multivariate Adaptive Regression Splines (MARS), Generalized Boosting Model (GBM), and Random Forest (RF).

**Figure 8:**
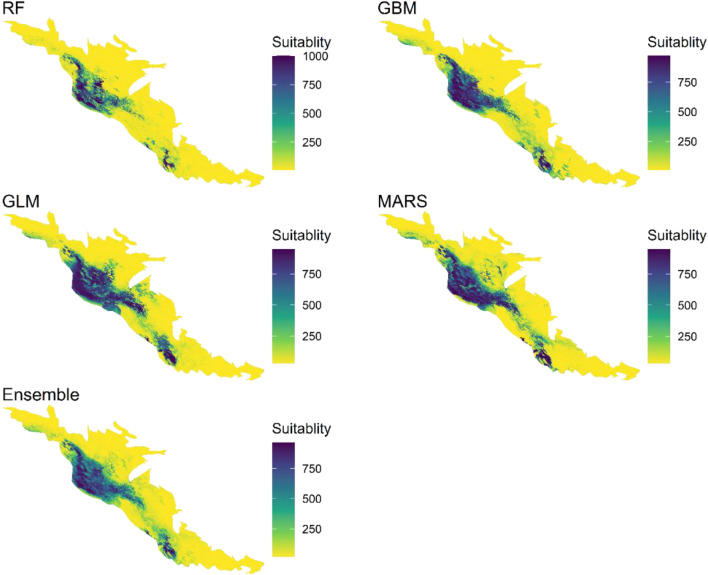
Model predictions of suitability generated using *O. persica* dataset at 1 km square scale in the western forests of Iran. Area modeled by Generalized Linear Model (GLM), Multivariate Adaptive Regression Splines (MARS), Generalized Boosting Model (GBM), and Random Forest (RF).

**Figure 9:**
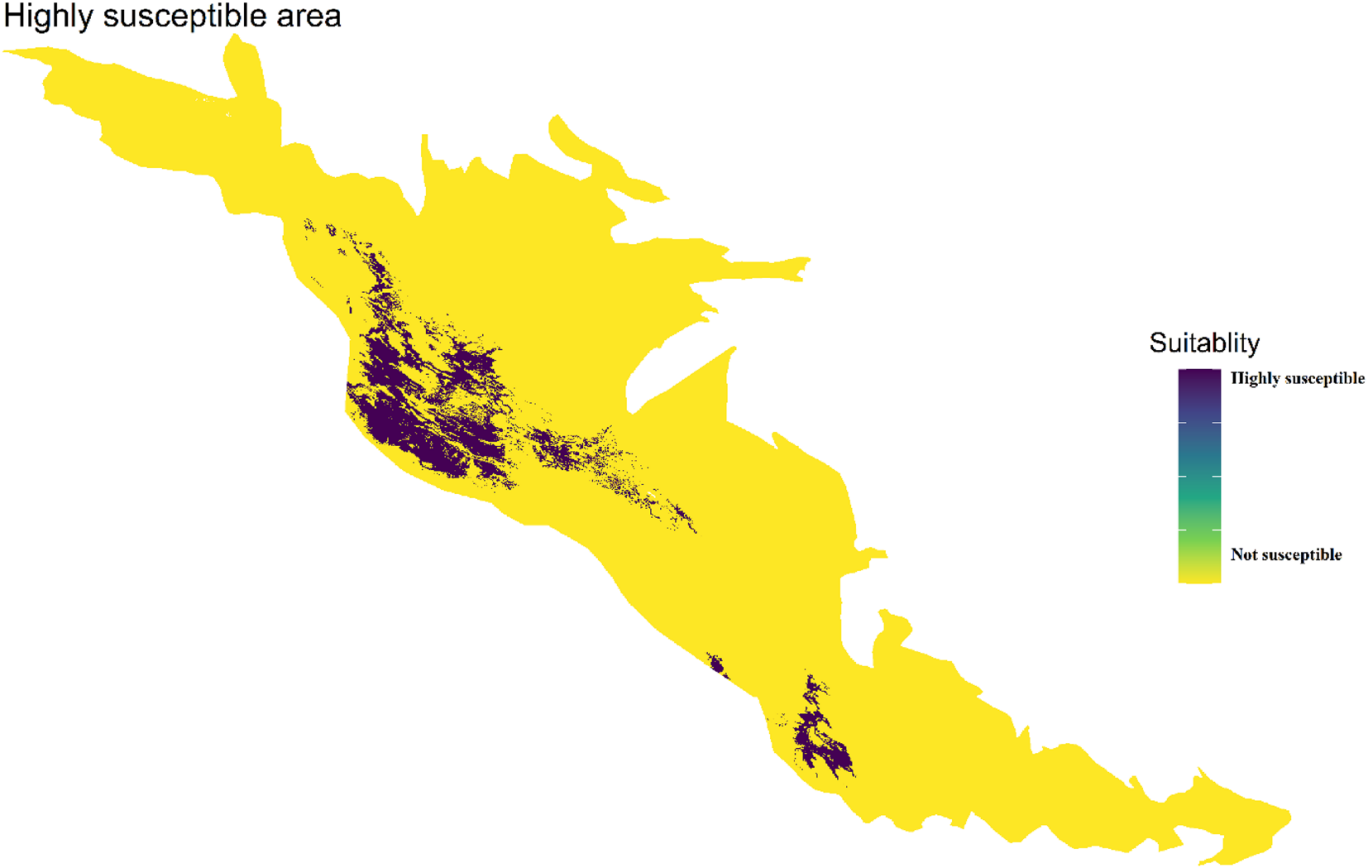
Final model predictions of suitability generated using the full CD dataset at 1 km square scale in the west forests of Iran. Area modeled by Generalized Linear Model (GLM), Multivariate Adaptive Regression Splines (MARS), Generalized Boosting Model (GBM), and Random Forest (RF).

Species distribution models using different scenarios based on global climate models (GCMs), are one of the methods to study climatic effects on disease distribution, present, and future habitat suitability, as well as characteristics of epidemiological diseases. As shown in Figs. 7, 8, 9, the northern Zagros forests (Lorestan, Ilam, Kermanshah oak forests) in addition to the southern Zagros forests (Fars and Kohgilouyeh va Boyer-Ahmad provinces) are more affected and capable of further epidemics. In all models, the median Zagros forests showed low to moderate suitability (the rest of the studied provinces which are listed in Supplementary material).

## Discussion

Oak charcoal disease (CD) causal agents, *B. mediterranea* (BM) and *O. persica* (OP) are of great concern due to devastating oak mortality in Iran^7,8^. A massive field survey indicated that there is a significant correlation between CD prevalence and precipitation regimes/temperature in the northern and southern Zagros oak forests, where current incidence and regions prone to epidemics indicate the occurrence of a definite crisis (Figs. 7, 8). The Middle East facing a precipitation shortage and ever-higher temperatures, especially in northern Turkey and Iran’s Zagros area^21,47,48^. The disease incidence variation and suitability area in the north and south of Zagros forests in comparison to the median parts could be due to the severe dust and water deficiency phenomenon continuously reported from those areas ^49–54^.

The effect of drought stress and wounding on the pathogenicity of BM and OP in greenhouse conditions on the seedlings of Persian oak (*Q. brantii*) showed that the presence of wounding is necessary to cause the disease. Also, according to Duncan's test, the difference in the average effect of the two levels of soil moisture was very significant (at the α = 0.01 level). With the increase of drought stress, the progression of necrosis in the stem tissue increased. Comparing the use of cuttings and saplings to study the pathogenicity of BM and OP isolates on Iranian oak (*Q. brantii*) showed that dark brown necrosis occurred in the subcutaneous tissue of cuttings and saplings. In both cases, the difference between the treatments and the controls was very significant (at the α = 0.01 level). Therefore, in the research related to CD and possibly other fungal diseases of oak trunks and branches, the cut branch can be used instead of stem manipulations. A very close relationship between drought and the sensitivity of oak species, especially *Q. brantii* to BM*,* was proved in the forest and greenhouse conditions. Based on previous reports in the oak decline pathosystem, fungal infections occur in healthy living trees as endophytes and then become invasive under water stress conditions. In most reports, BM has been reported to be aggressive on drought-stressed hosts^41^. Despite the tolerance of Persian oak species to the range of temperatures from − 31 to + 45 °C, the incidence of CD has increased dramatically in *Q. brantii* forests. Our results are in agreement with the studies conducted at regional scales^4,19,21–23^ where oak forests were surveyed for CD incidence and pathogenicity.

So far, SDMs (GLM, MARS, GBM, and RF) have not been used widely in forest disease epidemiology in Iran. SDMs have been defined as reliable models to predict the geographic distribution of rare species^55^. Albeit a total of selected 29 bioclimatic variables and applied models here were slightly different from those used in other local studies in CD distribution^4,23,56–60^, these parameters and models have shown high accuracy (ROC up to 96%) in CD modeling considering the extent of the study area, Zagros forests.

Jackknife analysis revealed that the environmental variable with the highest gain is, Bio18 (Precipitation of Warmest Quarter) which is the main important attribute in the distribution of OP, followed by, gsl, the most important parameter in the BM dispersal pattern, and is among five critical variables in the OP distribution (Fig. 4). The prolonged growing season is an indirect effect of a warming climate that has undeniable impacts on both host plants and their associated microbiota^61,62^. Faticov et al.^62^ showed a strong community shift in the foliar fungal community during one growing season in oak trees. As a part of the associated microbial community, from the beginning to the end of the season, the richness varied strongly among species of Ascomycetes fungi among other phyla, where most of them were endophytes/pathogens. According to our understanding and in agreement with the previous studies, the bioclimatic/environmental drivers will determine not only plant community dynamics but also hub taxa even on the same host plant species^63,64^. There is neither evidence of compositional microbial changes in the charcoal disease-oak pathosystem under changes in climatic factors over time and space nor extended growing season effects on the biology of CD's causal agents. Such research is necessary to uncover these network interactions in Iranian oak trees. Here we investigated the current status of BM and OP's potential distribution. Further studies aimed at determining their prevalence under optimistic and pessimistic scenarios by 2050 and 2100 are necessary.

Based on our pilot studies in the experimental phase (five pilots > 20 ha- Supplementary material in detail), the spread of the CD causal agents was not approved across the non-oak Zagros Forest trees. This claim needs more evidence since the association of the BM with wild almonds (*Amygdalus scoparia*) has been reported in this area^65^. Also, our study proposes measures such as enrichment of oak seeds and saplings, strengthening the soil and preventing its erosion, creating water catchment pits around the planted seedlings, and preventing fires to prevent the outbreak of pests and plant pathogens in the oak charcoal disease pathosystem. Management measures including avoiding wounding trees, maintaining good cultural practices such as: preventing or relieving compacted soil, and providing watering sources during hot, dry weather, pruning out dead or declining limbs may control the spread of the fungus on a tree, especially if combined with good cultural practices that increase the vigor of the tree and eliminate stresses. Infected wood should be destroyed immediately to keep the fungus from spreading, and severely infected trees should be cut and burned, Ideally, the stumps should be destroyed as well.

## Conclusion

Pathogenic fungi *Biscogniauxia mediterranea* and *Obolarina persica* cause significant losses in the Iranian oak forest in the north and southwestern. In the present study, pathogenic isolates were characterized all over oak forests in the Zagros habitat and their biological and ecological aspects have been determined. Among the variables tested, temperature and precipitation were the most important factors in determining the habitual variation of pathogenic fungi. As a result of a massive field survey with the implementation of bioclimatic/environmental variables in a high-accuracy machine-learning algorithms pipeline, the first national-scale distribution of the charcoal disease besides suitable areas that may be affected by ongoing epidemics has been provided. These maps can effectively be used in disease management strategies development. In addition, our work will have the potential to be used as a reference to ensure forest health and security in Iran.

### Supplementary Information

Supplementary Information.

## Data Availability

Data will be made available on request to the data analyzers, Kourosh Ahmadi and Meysam BakhshiGanje, who did the main modeling and biological aspects of the work.
