# Supplementary material for: Potential distribution of Biscogniauxia mediterranea and Obolarina persica causal agents of oak charcoal disease in Iran’s Zagros forests
Source: Sci Rep. 2024 Apr 2;14:7784. doi: 10.1038/s41598-024-57298-2 (PMC10987582; doi:10.1038/s41598-024-57298-2)
Supplement: Supplementary file 1 — Supplementary Information. [file 41598_2024_57298_MOESM1_ESM.docx]

**Supplementary material**

**Experimental and field assessments**

**Summary:**

In the context of climate change, the effects of prolonged or more severe droughts on pest and pathogen damage are a major concern for forest ecosystems. During 2013- 2015 the forest trees decline was observed, causing serious damage and the death of many trees. For this reason, the present study in the experimental phase aimed to have epidemiology assessments of oak charcoal disease throughout the western forest of Iran and the possibility of its control using useful methods on a pilot scale. The disease has been spread throughout the forests of Zagros which were extended from northwest to southwest of Iran covering approximately 3.1 M ha of oak forests. The infected tree species included *Q. brantii* (Persian oak) which has been dominated in all parts of Zagros mountains from north to south, especially on the southern side of the mountain including Ilam, Lorestan, Kohgilouyeh va Boyer-Ahmad, Fars, and Kermanshah provinces. The signs of the disease are the deterioration and death of trees, the browning and untimely fall of the general landscape of the part of the forest where the disease is common, the oozing of white gum on the branches at the beginning of the infection, and the release of a large amount of dark gum from the trunks of old trees. In the fall and the following winter, most of the affected trees disappeared and charred marks appeared. Large cankers carrying a shiny black or matte layer of fungal stroma on the surface of the wood, which is the name of this disease, were evident on the trees. Based on morphological characteristics and the ITS region sequencing (Internal transcribed spacer), the fungi were identified as *Biscogniauxia mediterranea* (BM) and *Obolarina persica* (OP). Pathogenicity tests were conducted using an isolate of *B. mediterranea* and *O. persica* on six-month-old *Q. brantii* seedlings using a mycelial plug of both agents colonized potato-dextrose agar and the symptoms were observed after two months and the same fungus was re-isolated. The required data of wood-boring beetles’ association with charcoal disease epidemy were obtained by direct observation and field sampling. The samples were collected based on a systematic sampling pattern in each plot. The borer beetles were collected by splitting the dried branches, cutting the trunk and branches and keeping them in proper condition. The results showed that the beetles are from *Cerambycidae* and *Buprestidae* families including; *Chalcophorella bagdadiensis, Agrilus hastulifer, Chrysobotris parvipuncta*. Seedlings of *Q. brantii* were put under different treatments. The experiment was a 2 × 3 randomized complete block design with three replications of eight seedlings per treatment. The treatment combinations were two levels of irrigation and inoculation with two types of pathogens: watering was based on 100% field capacity without fungal inoculation; watering based on 100% field capacity and inoculated with the OP; watering based on 100% field capacity and inoculated with the BM; watering based on 20% field capacity without fungal inoculation; watering by 20% field capacity and inoculated with the OP; and watering by 20% field capacity and inoculated with the BM. Each treatment combination had three replicates of eight seedlings.

According to Duncan's test, the difference in the average effect of the two levels of soil moisture was very significant (at the α = 0.01 level). With the increase of drought stress, the progression of necrosis in the stem tissue increased. Based on field driven-data, our management guidelines propose a mixture of measurements including elimination of severely infected trees; proper pruning treatments; enrichment of oak seeds and saplings, strengthening the soil and preventing its erosion, creating water catchment pits around the planted seedlings, and preventing fires.

**Charcoal Disease etiological assessments by location:**

**L1- Ilam province**

The co-occurrence of BM and OP in field observations was documented in 3 out of 16 surveyed regions (pilot No. 1, Anarak and Dalahoo) during two successive years in Ilam province. 13 other regions showed a spectrum of infections, by BM or OP. The slop and height were determined, as the most affecting drivers in pilot surveys so that all contaminated samples in pilot 1 were in the slope of 15-30% and in terms of altitude 30% of the samples were in the altitude of 1480 to 1520 m and 70% of the contaminated samples were located in the altitude of 1520-1600 m. In addition, all contaminated samples in pilot No. 2 were in the slope class of 0-15% and in terms of altitude, 30% of the samples were at 1840 to 1860 m, 35% at 1860 to 1880 m, 27% at 1880 to 1900 m and 8% were located at an altitude of 1900 meters above sea level. The association of wood-boring beetles with infected trees was estimated as 35% and 68% in pilot numbers 1 and 2, respectively. Frequent sampling from the protected area of Chogha Sabz Forest Park (2013-2015), showed a high level of infection with CD and wood borer beetles association. The percentage of infection in this area was estimated at 26%, indicating the devastating impact of the disease even in protected areas. Another goal of the field experiment was to obtain the rate of progression and transmission of the disease from infected trees to healthy ones, and therefore the health status of several healthy-symptomless trees was considered. After two years, 26% and 26.31% of progression and transmission rates were estimated in pilots 1 and 2, respectively. Also, using the Transect sampling method, the disease severity was determined as 64.125 and 49.5 for both pilots 1 and 2. Estimating the relationship between the DBH and the infection rate in the two pilots, the least amount of damage was recorded for young trees with DBH < 10 cm in comparison to well-established trees with DBH> 50 cm in the second pilot (55% vs 13 % infection rate). The average DBH of dead trees was 31.5 and 39 cm respectively in pilots 1 and 2. The diameter of the insect's outlet hole (in two ranges of >.5 and <.5 cm) of the causal agent's infection rates were investigated and inconsistent results between the two pilots were obtained.

**L2- Kermanshah province**

With almost 527000 ha (Mainly covered by *Q. brantii* and minor populations of *Q. infectoria*), the Kermanshah forests continue across the mountains from northwest to south and then southwest. A population of a hundred high-forest oak trees was selected in an area of about 1.5 hectares in the Kale-Zard region where the percentage of oak trees with CD symptoms was relatively high. In three years (from 2013 to 2015) after the initial examination (screening the health status of trees), the number of trees on which stroma developed was calculated and used as an indicator of disease progression. The emergence of new stroma on trees that were already marked as infected was also examined. BM and OP stroma did not appear on trees that were screened as healthy at the time of sampling although they were located adjacent to diseased trees. The results of studies in Kermanshah province showed that in all forest areas of the province, a spectrum of the complications of oak withering like declining single trees (more than 5% of foliage withering) or completely dry with signs of CD (black stroma containing ascocarp of BM or OP), scattered among seemingly healthy trees with a relatively low percentage (approximately 2%) or nonexistent diseased oaks have existed. However, in some areas, this complication has appeared in the form of spots and with high intensity, so the percentage of complete dryness or severe decline of oak trees in these spots varies from 5 to 40%. The percentage of trees with signs of CD was estimated to range from almost zero up to 20%. These spots include regions with almost 2-3 hectares and are mainly located on the southern slopes. The association of wood-boring beetles with diseased trees was demonstrated, except for the Ghamrali region in Guilane-Gharb county of Kermanshah province. The co-occurrence of BM and OP with diseased trees was documented in Kale-Zard and Ghalache (2 out of 16).

**L3- Kohgilouyeh va Boyer-Ahmad province**

Oak trees comprise nearly 80% of the forest within Kohgiluyeh va Boyer-Ahmad province in a way that most dense/ha oak populations are located in this region of Zagros' habitat. Two pilots (10 hectares) were selected with one hundred trees in coppice form masses (lifespan of 10 to 25 years), one in Basht city (tropical region) and the other one in Kushk region in Boyer-Ahmad city (cold region). In both tropical pilots, only BM was isolated and identified as the causal agent. The infection rate of CD was 62% in the Basht pilot and 54% in the Boyer Ahmad pilot, respectively. There was a significant relationship between disease incidence and wood-boring beetles association (beetles of *Cerambycidae* and *Buprestidae* families) in which 83% and 81% co-occurrence were determined, respectively, in pilots 1 and 2. Slow and gradual disease incidence was observed in 20 to 25 20-year-old trees, while the infection in young trees occurred faster. In both pilots, the oak infection rates in the valleys were less than those located on the slopes, which could probably be attributed to the low water availability for roots and the shallow depth of the permeable soil.

**L4- Fars Province**

In this province, BM was determined as the most prevalent CD causal agent. The main infected areas were reported as Dashte-Barm, Dorahi-Kalani (near Kazeroun county), followed by Firoozabad at the end of the Zagros forests in the west of the country. The severity of the disease was estimated at low rates in the forests of Saranjalak, Mamo and Alaqshkari in NourAbad-Mamasani and the. In the Dashte-Barm region, obvious damage was observed accompanied by wood-boring beetles, while in Komare-Sorkhi with a rare invasive insect’s association rate, the severity and symptoms of the disease were limited.

**L5- Lorestan province**

Although the distribution of the BM was higher than the OP, both species were observed simultaneously in some regions, such as the Bishe area. Termites co-damage was significant in Ghale-Nasir and Pol-Dokhtar. Regardless of the damage caused by wood-boring insects, damage induced by the disease was significant in Kouhdasht and Shourab of Khorramabad. Significant disease losses were reported in one region (Sarnouh-Blouzan) after firing on coppice oaks. The mortality rate in the studied areas varied from 0.5 to 6 in the first year to 2-8% in the second year. The highest mortality was observed in the areas of Doroud (Sper Valley), Bishe, and Razan. Results indicated the wider distribution range of OP in the forests of eastern Lorestan.

**L6- Hamedan province**

The forests of Gyan, Zarrin Bagh, and Siah Darreh (almost 250 hectares) are the only natural forests of Hamedan province that remain as spots in the western oak forests of Zagros' habitat. Only BM was characterized by diseased trees (dominated by high forest trees). The infection rate of oak trees was determined as 5, 1, and 2%, respectively. In addition to wood-boring beetles, the presence of oak gale bees, as well as hawthorn mites, is widespread in these forest areas. With the progress of the drying process over 3-5 years, the complete decline of oak trees was finally observed.

**L7- Kurdistan province**

Delivered samples from infected oak trees from Marivan County were examined concerning the present assessment of CD causal agents and results showed that both BM and OP are associated with diseased trees.

**L8- Khuzestan province**

The association of wood-boring beetles-BM with diseased oak trees in different parts of Khuzestan province, southwest Iran, has been documented.

**Figures legend**

**Appendix 1. A-**Symptoms of charcoal disease and the presence of causal agent’s stroma on oak trunks, **B, C-** Formation of new fungal stroma on oak tree, **D, E-** Pathogenicity of an isolate of *B. mediterranea* and *O. persica* on one-year-old seedlings of Iranian oak (*Quercus berantii*), respectively., **F-** The place where the beetle enters the trunk of the oak tree.

**Appendix 2.** The cut trunks of dried oak trees infected with charcoal disease, Khorramabad, Iran

**Appendix 3. A-** Production of new year's branches resulting from cutting the upper parts infected with charcoal disease causal agents on the oak tree, **B-** Comparison of successful pruning (right) and unsuccessful pruning (left)

**Appendix 4.** The multiplication signs refer to Kermanshah, Ilam 1, Ilam 2, Kohgilouyeh- va Boyer Ahmad 1, and Kohgilouyeh- va Boyer Ahmad 2 experimental pilots from northern to southern within the study area, respectively.


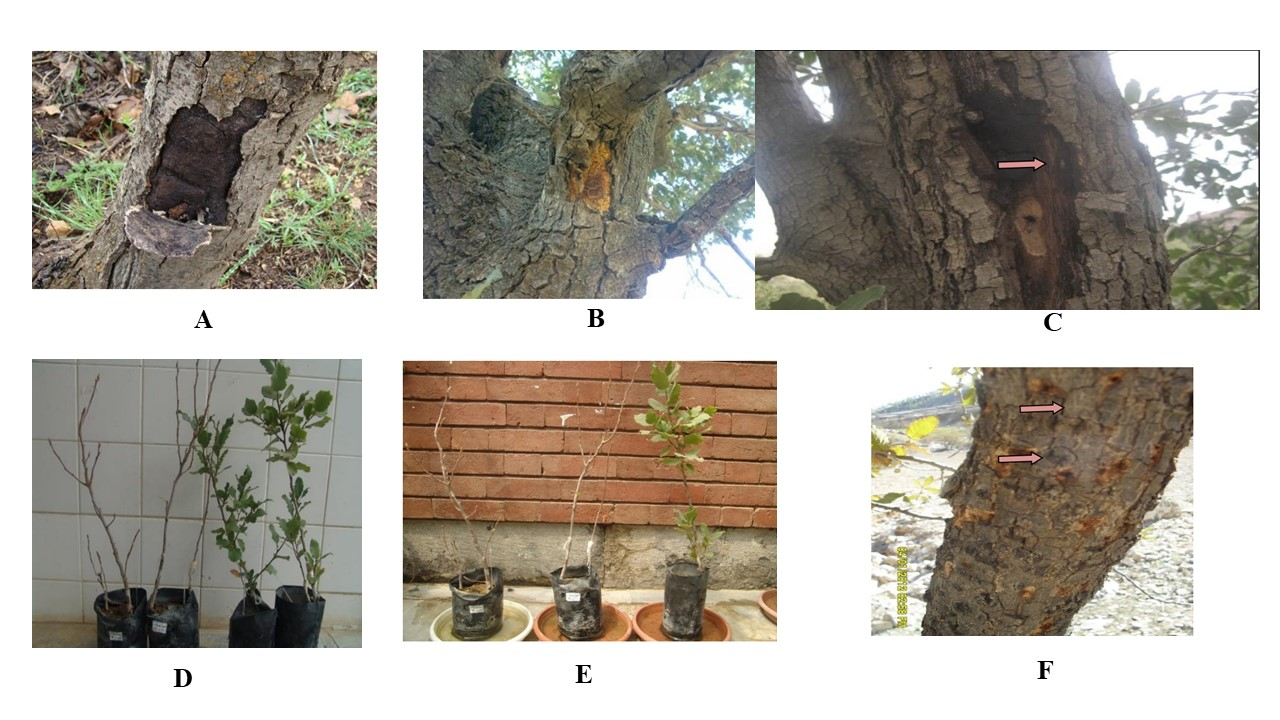


**Supplementary material 1. A-**Symptoms of charcoal disease and the presence of causal agent’s stroma on oak trunks, **B, C-** Formation of new fungal stroma on oak tree, **D, E-** Pathogenicity of an isolate of *B. mediterranea* and *O. persica* on one-year-old seedlings of Iranian oak (*Quercus berantii*), respectively., **F-** The place where the beetle enters the trunk of the oak tree.


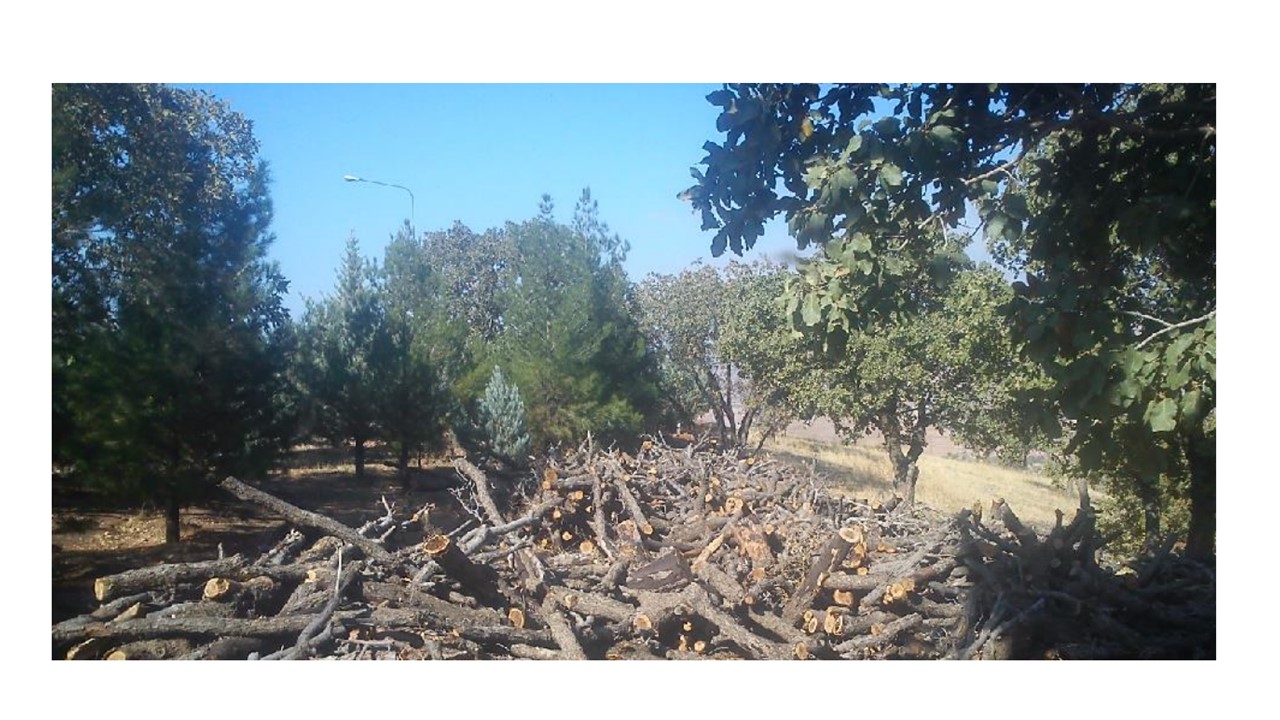


**Supplementary material 2.** The cut trunks of dried oak trees infected with charcoal disease, Khorramabad, Iran


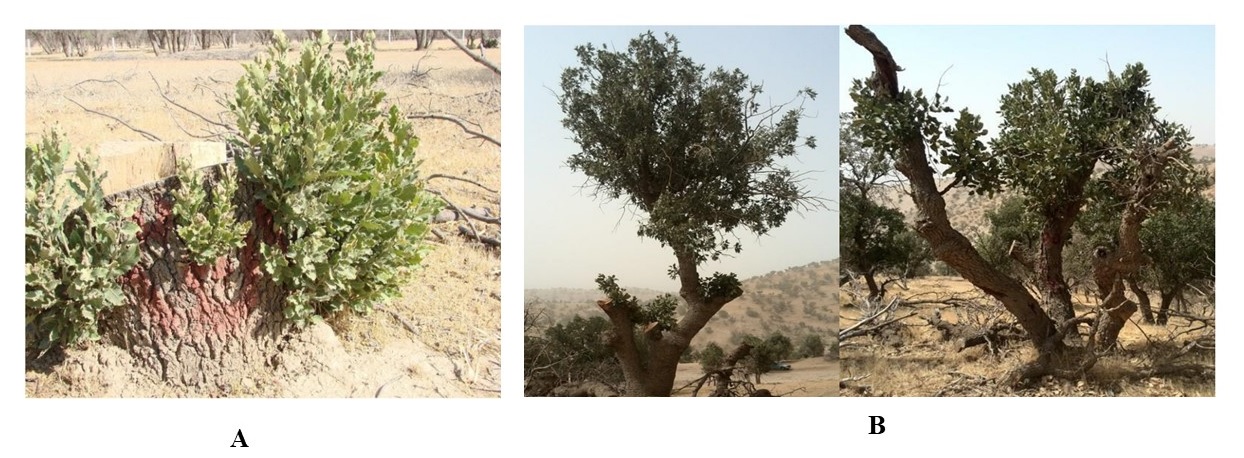
 **Supplementary material 3. A-** Production of new year's branches resulting from cutting the upper parts infected with charcoal disease causal agents on the oak tree, **B-** Comparison of successful pruning (right) and unsuccessful pruning (left)


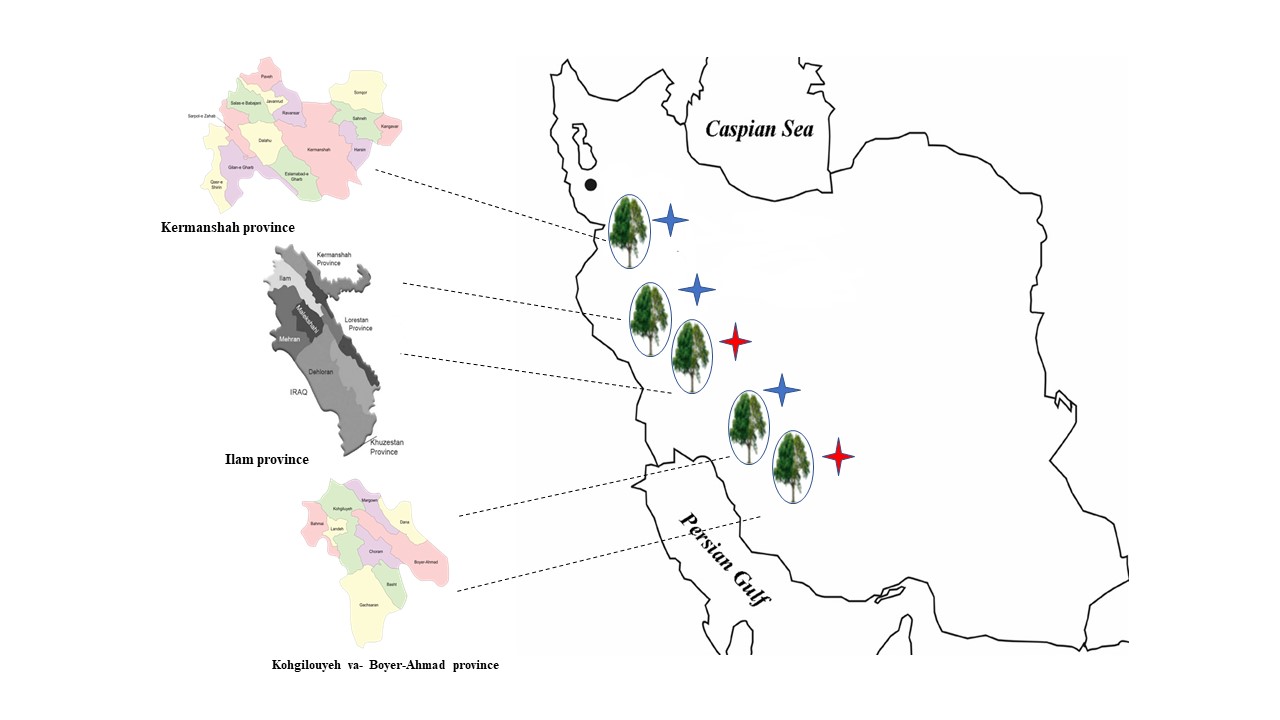


**Supplementary material 4.** The multiplication signs refer to Kermanshah, Ilam 1, Ilam 2, Kohgilouyeh- va Boyer Ahmad 1, Kohgilouyeh- va Boyer Ahmad 2 experimental pilots from northern to southern within the study area, respectively.
